# Occurence and Bioactivities of Funicone-Related Compounds

**DOI:** 10.3390/ijms10041430

**Published:** 2009-03-30

**Authors:** Rosario Nicoletti, Emiliano Manzo, Maria Letizia Ciavatta

**Affiliations:** 1 Council for Research and Experimentation in Agriculture, C.A.T. Research Unit / Via Vitiello 108, Scafati 84018, Italy; 2 Institute of Biomolecular Chemistry, National Research Council / Via Campi Flegrei 34, Pozzuoli 80078, Italy; E-Mails: emiliano.manzo@icb.cnr.it (E.M.); letizia.ciavatta@icb.cnr.it (M.C.)

**Keywords:** Biological activity, chemotaxonomy, fungal extrolites, funicones, Penicillium

## Abstract

Studies on production of secondary metabolites by fungi have received a substantial boost lately, particularly with reference to applications of their biological properties in human medicine. Funicones represent a series of related compounds for which there is accumulating evidence supporting their possible use as pharmaceuticals. This paper provides a review on the current status of knowledge on these fungal extrolites, with special reference to aspects concerning their molecular structures and biological activities.

## 1. Introduction

Starting with the discovery of funicone [1], a series of structurally related compounds have been found to be produced by a number of fungal species, particularly within the anamorphic genus *Penicillium*, which is quite famous for its production of antibiotics and other compounds that have undergone development as pharmaceuticals. The observation of notable biological properties has recently increased the interest of researchers in finding new sources and structural variants of this extrolite, whose occurrence within more or less closely related species may also provide useful information with reference to chemotaxonomic implications. Aspects concerning production, characterization and biological activity of funicone-related compounds are reviewed in this paper.

## 2. Molecular Structures and Producing Species

**Figure 1:**
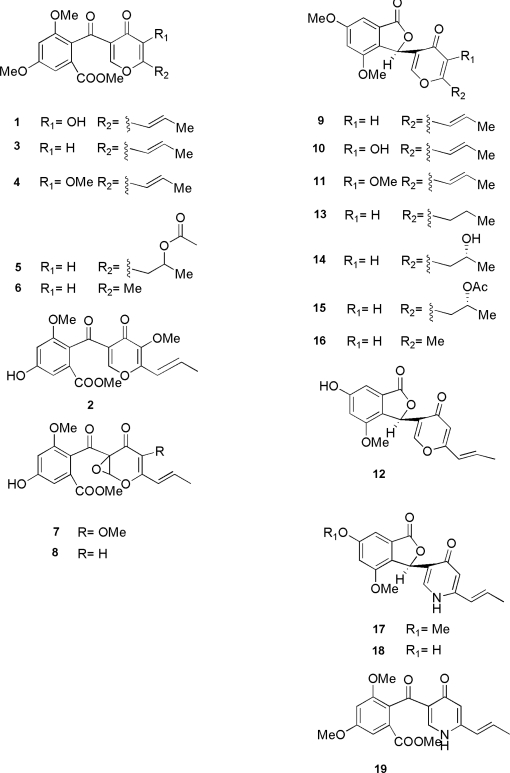
Molecular structures of funicone-related compounds.

The structures of funicone-related compounds (Figure 1) are based on a γ-pyrone ring which is linked through a keto function to a β-resorcylic acid nucleus whose carboxylic group is esterified by methanol; depending on the specific metabolite, one or more hydroxyl groups may be methylated, while several substitutions occur at the carbon atoms of the γ-pyrone nucleus. The parent molecule of the series is funicone (**1**) [benzoic acid, 2-[[5-hydroxy-4-oxo-6-(1*E*)-1-propenyl-4H-pyran-3-yl]carbonyl]-3,5-dimethoxy, methyl ester] [1], which was named after the Latin name of the producing organism, *Penicillium funiculosum*, a species belonging to the subgenus *Biverticillium* [2]. However, considering its description and morphological appearance [3], the producing strain should probably be more correctly ascribed to *Penicillium pinophilum*, a species that was later neotipified and separated by *P. funiculosum* [4]. The compound has been more recently detected in a strain of *Talaromyces flavus* [5], an ascomycetous mycoparasite (*Eurotiomycetes, Eurotiales*) whose anamorphic state (*Penicillium dangeardii*, syn. *P. vermiculatum*) is taxonomically closely related to species in the subgenus *Biverticillium*, and in *Penicillium simplicissimum* (subgenus *Furcatum*) [6].

Some funicone-related products differ by just one or few substitutions on this fundamental molecular frame. This is the case of isofunicone (**2**), a structural isomer produced by an unidentified *Penicillium* strain [7], in which a methoxyl group on the aryl moiety is interchanged with the hydroxyl group on the γ-pyrone nucleus. The latter is absent in deoxyfunicone (**3**), characterized in two different unidentified *Penicillium* isolates [8,9], and later also detected in *T. flavus* [10] and *Penicillium citreonigrum* (teleomorph *Eupenicillium euglaucum*, subgenus *Aspergilloides*) [11]. Conversely, the hydroxyl group is methylated in 3-*O*-methylfunicone (**4**), produced by a soil strain of *P. pinophilum* [12]. Small amounts of the latter substance (funicone methyl ether) had been previously obtained synthetically by treating funicone with excess diazomethane in dichloromethane [1].

Substitutions concerning the propenyl tail occur in other compounds. In actofunicone (**5**), again isolated from *T. flavus* [10], this function is replaced by an acetoxypropyl residue, while it is substituted by a methyl group in rapicone (**6**), produced by the anamorphic species *Ramichloridium apiculatum*, an antagonist of plant pathogenic fungi known as powdery mildews (*Erysiphaceae*) that is taxonomically unrelated to *Penicillium* [13]. Derivatives bearing an epoxide function on the γ-pyrone ring (**7–8**) have been found for both 3-*O*-methylfunicone [14] and deoxyfunicone [5], respectively, from strains of *P. pinophilum* and *T. flavus*; as the latter compound is actually an isomer of funicone, it may represent a possible intermediate in its biosynthesis.

Vermistatin (**9**) [1(3H)-isobenzofuranone, 4,6-dimethoxy-3-[4-oxo-6-[(1*E*)-1-propen-1-yl-4H-pyran-3-yl], (3*R*)], produced by *T. flavus* [5,10,15–17], presents a slightly more complex molecular structure where it can be supposed that, after being reduced, the ketide group interposed between the two subunits reacted with the carboxymethyl function of the resorcylic nucleus, to give a benzophthalide moiety that has some relevance for the biological activity of the compound. The same molecule has been later extracted and characterized under the name of fijiensin by *Mycosphaerella fijiensis*, an ascomycetous fungus (*Dothideomycetes, Capnodiales*) known as the agent of ‘black sigatoka’, a destructive disease of banana plantations [18]. More recently production of vermistatin has been documented by another biverticillate species, *Penicillium verruculosum* [19], by the already mentioned *P. simplicissimum* [6] and *P. citreonigrum* [11], and by the novel species *Talaromyces thailandiasis* [20]. Finally, its finding as an extrolite of an endophytic ascomycetous strain, isolated by a mangrove (*Kandelia candel*) in Hong Kong and identified as *Guignardia* sp. (*Dothideomycetes, Botryosphaeriales*) [21], confirms that the biosynthetic ability by fungi for this particular compound is possibly even more widespread than currently known. Vermistatin is often extracted together with some derivatives, such as hydroxy- and methoxyvermistatin (**10–11**) [21], 6-demethylvermistatin (**12**) [22], dihydrovermistatin (**13**), hydroxy- and acetoxy-dihydrovermistatin (**14–15**), and penisimplicissin (**16**) [6,20]; in analogy with the difference occurring between funicone and rapicone, the latter presents a methyl group replacing the propenyl tail. It is interesting to note that another variant corresponding to a dihydro-derivative of compound (**10**), was also artificially obtained by acid reduction of funicone under zinc catalysis, possibly in consequence of the conversion of the *ortho* carbonyl to a secondary alcohol and ensuing ring closure with formation of the phthalide lactone [1]. This finding provides evidence that vermistatins may be elaborated by enzymes working on precursors with a funicone skeleton.

**Figure 2:**
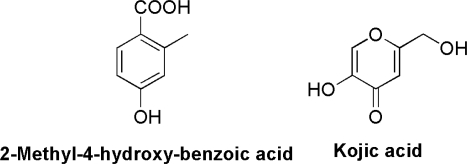
Molecular structures of two possible precursors of funicone-related compounds.

Merlini *et al.* [1] consider biosynthesis of funicone to occur following a polyketide pathway through a hypothetical tricyclic intermediate. This opinion is shared for the formation of deoxyfunicone [8] and rapicone [13], in the latter case by considering analogies with other fungal heptaketides. Actually, the presence in the culture filtrates of different strains of *T. flavus* of other polyketides possibly sharing part of a common biosynthetic pathway, such as vermiculin, vermiculinic acid and compound NG-012 [10,23], corroborates this assumption. However, the finding of 2-methyl-4-hydroxybenzoic acid (Figure 2), particularly abundant in the mycelial cake developed in liquid cultures of *P. pinophilum*, has stimulated a hypothesis that 3-*O*-methylfunicone could rather be directly assembled by two molecules of such compound undergoing oxidation [24]. Reactions carried out in artificial conditions [25] indicate that another possible precursor involved in the biosynthesis of the γ-pyrone moiety of funicones is kojic acid (Figure 2), a compound that is also reported as a fungal extrolite, particularly in *Penicillium* and *Aspergillus* species [26,27].

Very recently, another series of funicone-related alkaloids has been extracted from semi-solid cultures of an endophytic *Penicillium* strain recovered from *Quercus variabilis*, whose morphological description corresponds to a biverticillate species related but not identical to either *P. pinophilum* or *P. verruculosum*. Penicidones A-B-C (**17–18–19**) are structural analogues of vermistatin, 6-demethylvermistatin and deoxyfunicone, respectively, in which the heterocyclic moiety consists of a quite unusual γ-pyridone nucleus [28], that to our knowledge can be only found in another series of *Penicillium* extrolites, the citridones [29]. It is thought that the N atom in the pyridone ring could originate from glutamate under the catalysis of aminotransferase [28], but pyridones have been also synthesized as hydrophilic derivatives of kojic acid [27]. Moreover, a pyridone variant was also artificially obtained by hydroxy-dihydrovermistatin reacting with methylamine in methanol [1]. Therefore, the evidence is strong that penicidones and funicones be synthesized through a common biosynthetic pathway.

Funicone-related compounds represent a homogeneous group of fungal metabolites sharing a number of physico-chemical features (Table 1). They are water insoluble, therefore readily extracted by most organic solvents, with the exception of *n*-hexane; they confer a yellow color to the solution, while appearing as white or pale yellow powders when dried. An exception is represented by actofunicone, that is the only member of the series possessing an oily consistency at room temperature. Melting points of the other products are in a range between 128°C (deoxyfunicone) and 227°C (hydroxyvermistatin). The presence of an aromatic ring highly conjugated with the double bonds of the γ-pyrone moiety is responsible for UV-absorbance, whose values can be relevant in view of a rapid detection in the culture extracts.

## 3. Methodology for Production and Extraction of Funicone-Related Compounds

Besides the previously mentioned analogy existing between the penicidones and three ‘pyronic’ funicones, by considering the fundamental difference in the aryl moieties of funicone and vermistatin it results that substitutions occurring at the γ-pyrone ring generate several pairs of corresponding analogues. This is the case of funicone and hydroxyvermistatin, deoxyfunicone and vermistatin, 3-O-methylfunicone and methoxyvermistatin, rapicone and penisimplicissin. It may be questionable if some of these variants are eventually artefacts ensuing treatment with organic solvents, particularly methanol. Actually, the fact that they can be recovered by different strains and by means of different methodologies for extraction and purification seems to contradict such a hypothesis. However, as conditions for culturing the fungal strains and/or the extraction of culture filtrates may somehow influence the biosynthetic process and/or the recovery of the fermentation products, it is quite important to consider these aspects by comparing the procedures followed for each compound of the series.

Information concerning composition of the substrates used for culturing the producing strains is provided in the appendix, while growth parameters are summarized in Table 2, together with conditions for extraction and purification. The fact that these compounds are produced on a variety of substrates, more or less rich in nutrients, indicates that their production is possibly constitutive rather than being induced by particular carbon sources or other components.

However, specific assays carried out for vermistatin have shown that the nitrogen source and the availability of some organic acids, such as malate and succinate, may notably influence the yield [30,31], which otherwise basically depends on duration of culturing. In most cases cultures are maintained for three weeks or longer, but available data are not sufficient to indicate the most appropriate length of the fermentation cycle. Both funicone and deoxyfunicone were initially recovered after just 4–6 days of growth of the producing strains [1,8], but their finding as major products after prolonged culturing [5,6,9] contrasts the eventuality that they may actually represent intermediates in the biosynthesis of other compounds of the series. Funicone was obtained in a comparatively higher yield by culturing the producing strain on moist barley grains, and extracting the cultures twice: first with a mixture of dichloromethane and methanol (1:1), then with ethyl acetate after re-suspending the residue in water [5]; purification of the compound together with small quantities of 9,14-epoxy-11-deoxyfunicone and vermistatin was achieved by a multi-step chromatographic procedure that may be quite helpful with the aim to recover by-products eventually produced in small quantities. A more complex methodology leading to the extraction of several analogues [10] consisted in a preliminary extraction with acetone, followed by a further extraction with ethyl acetate; the resulting oily material was dissolved in methanol and submitted to MPLC eluting with a linear gradient from 30% to 80% acetonitrile; the active fractions, containing deoxyfunicone, actofunicone and vermistatin, were then purified through HPLC.

Vermistatin can be also considered a terminal product of the biosynthetic process; in fact, besides having been recovered after just 1-week culturing [10,15], most methods provide for its extraction after a prolonged fermentation [5,6,11,17,19–21]. In the procedure set up for extraction by *M. fijiensis* [18] the compound could be detected no earlier than 21–24 days, indicating that particular carbon sources and the presence of light may depress its biosynthesis.

## 4. Biological Activities of Funicone-Related Compounds

So far biological activity of the known funicone-related compounds has not been studied comprehensively due to the different objectives pursued by several independent investigators. At first funicone did not show any notable biological effects, as it was found to just slightly stimulate rooting of tomato sprouts, while assays carried out against *Bacillus subtilis* and *Staphylococcus aureus* resulted in quite poor antibiotic properties [3]. More recently, fungitoxicity was observed in assays carried out by a paper disc method against the human pathogenic species *Aspergillus fumigatus*, while two yeasts of medical relevance, *Candida albicans* and *Cryptococcus neoformans*, were unaffected [5]. The same study showed a low fungitoxic activity by 9,14-epoxy-11-deoxyfunicone against *Aspergillus niger*, while the homologous compound 3-*O*-methyl-5,6-epoxyfunicone was found to be inactive against *Rhizoctonia solani, Alternaria alternata* and *Fusarium solani*, possibly due to structural unstableness [14]. In an *in vitro* assay developed for screening new potential herbicide products, isofunicone inhibited the germination tube of pollen grains of *Camellia sinensis* at a concentration of 10 μg/mL [7]. Rather than being phytotoxic, deoxyfunicone showed plant growth stimulatory properties on radicles of lettuce and Chinese cabbage seedlings at concentrations between 10 and 50 μg/mL; moreover the compound displayed notable fungitoxicity against *Gibberella fujikuroi*, *Pyricularia oryzae* and *F. solani* f.sp. *phaseoli*, while no antibacterial effect was evident [8]. As assayed for insecticidal properties against Lepidoptera larvae (*Spodoptera littoralis*), both deoxyfunicone and vermistatin were inactive, while a very low activity was recorded against *Artemia salina* [11]. A more considerable pharmaceutical application of deoxyfunicone may derive on account of the antiviral properties disclosed as a HIV-1-integrase inhibitor [9,32].

Consistent fungitoxic properties have been also observed for 3-*O*-methylfunicone, that suppressed *in vitro* mycelial growth of a number of plant pathogenic fungi, such as *R. solani, A. alternata, Cylindrocladium scoparium* and *F. solani*, at a concentration of 100 μg/mL [12,33]. The same concentration also inhibited dermatophytic species, such as *Trichophyton rubrum* and *Microsporum canis*, while, as already pointed out for other compounds of the series, it was not effective against *C. albicans* [34]. Antiproliferative properties by 3-*O*-methylfunicone have later resulted against human tumor cell lines. In fact, cytostatic effects and the induction of programmed death were observed on HEp-2 cells (derived from larynx carcinoma) at a concentration of 60 μg/ml [35]. Results of biological assays were quite similar on HeLa cells (cervix-uteri carcinoma), which are arrested at the G1 phase of the cell cycle and undergo apoptosis following a p53 independent pathway in consequence of the activation of pro-apoptotic genes (p21) whose expression reflects the inhibition of the Cdk4-cyclin D1 complex [36]. Antiproliferative and pro-apoptotic properties have been also evidenced on other tumor cell types derived from lung carcinoma (A549) [37], and melanoma (A375P and A375M), where the inhibitory effect on cell cycle progression occurs at the G2 boundary, with a reduction in the expression of cyclin B1 and cyclin-dependent kinase p34 [38]. Moreover, the compound has been found to inhibit the gene expression of typical markers of tumor progression, such as survivin and human telomerase reverse transcriptase (h-TERT), and to strongly affect cell proliferation and motility of breast cancer MCF-7 cells by down-regulating αvβ5 integrin and inhibiting matrix metalloproteinase (MMP-9) secretion. This effect is selective, as it was not observed on a non-tumor breast cell line (MCF-10) [39]. Inhibition of cell motility is also associated to modifications in cell shape and in the distribution of tubulin fibers of MCF-7 cells. The latter effect may depend on the trimethoxylated aryl moiety, that brings some funicone compounds in functional analogy with other natural products well-known as antitumor pharmaceuticals, such as combretastatin, the podophyllotoxins, and the chalcones [40].

Some level of cytotoxic activity by a funicone-related compound had been previously demonstrated for vermistatin on murine leukemic cells (P388) and Ehrlich ascites, where the compound acts as a RNA-synthesis inhibitor [15]. Possible relevance of vermistatin as an antitumor compound has been pointed out more recently, after its inhibitory properties were observed against mouse lymphoma cells (L5178Y) at a concentration of 10 μg/mL; moreover the compound was slightly inhibitory toward several kinases, such as aurora A and B, cdk 4/cyclin D1, the insulin-like growth factor receptor-1, ErbB2, BRAF-VE, Akt1 and the vascular endothelial growth factor receptor-2, involved in the cell cycle progression and apoptosis induction, or implicated in the pathologic angiogenesis associated with tumor growth [11]. Otherwise, quite a low biological activity has been reported on different organisms, starting with its effects as a banana-specific toxin [18]. As assayed against *A. fumigatus* and *A. niger*, vermistatin and its derivatives showed almost no antifungal activity [6]. Together with actofunicone and deoxyfunicone, it has also proved to be ineffective against *C. albicans* at concentrations up to 300 μg/mL; however, these compounds became inhibitory when administered in association with myconazole [10]. As this fungicide is frequently ineffective by itself on immunocompromised patients, the synergistic capacity of funicones to potentiate its efficacy by 5-10 times provides more indications for their chemotherapeutic potential. Finally, further pharmaceutical perspectives of vermistatin can be considered in view of the anxiolytic effects shown by other compounds with a benzalphthalide skeleton [41]. Actually, the phthalide moiety seems to be quite important for biological activity, as some derivatives obtained by artificial hydrogenation proved to be inactive when modifications were introduced in this part of the molecule [42]. However, the inactivity observed in the case of dihydrovermistatin [11] suggests that the propenyl tail is also quite relevant to this regard. The higher cytotoxic activity of methoxyvermistatin, as measured against KB and KBv200 cells (derived from epithelial carcinoma) [21], also indicates that a remarkable importance pertains to the methoxyl group at the γ-pyrone ring, which also characterizes 3-O-methylfunicone. To this regard, it must be considered that the antifungal activity reported for several derivatives of kojic acid [43] provides a further indirect evidence that biological properties of funicone-related compounds are in part dependent on the γ-pyrone moiety.

By reason of their very recent discovery, so far penicidones have been just preliminarily assayed for inhibitory properties against a few cell lines, such as HeLa, KB, K562 (myeloid leukemia) and SW1116 (colon cancer), displaying moderate cytotoxicity [28]; the comparative effects of these extrolites and their γ-pyrone analogues should be more thoroughly considered in order to obtain further evidences concerning the active site of funicone-related compounds.

## 4. Conclusions

As introduced above, the quite notable biological activities of funicone-related compounds put forward a perspective for their development as pharmaceuticals. Therefore it is likely that these extrolites will be the subject of further investigations for production by fungi in the near future, also considering that the finding of a number of analogues in several unrelated species belonging to two different classes introduces a possible more widespread occurrence. For the time being most reports concern *Penicillium* species, particularly those ascribed to the subgenus *Biverticillium* and their related *Talaromyces* teleomorphs [34,44,45]. Therefore, following the consolidated approach established for taxonomy of the terverticillate *Penicillia* (subgenus *Penicillium*) [46], the increasing amount of data gathered within this context is susceptible to be considered for chemotaxonomic purposes, particularly in view of attaining to a more accomplished characterization of a number of biverticillate species whose taxonomic status is uncertain [4,44,45]. In this regard, methods allowing a rapid analysis of fungal extracts, particularly those based on HPLC [11,23,44], are likely to provide a substantial contribution for a more considerable detection of these compounds.

## Figures and Tables

**Table 1:** Main physico-chemical features of funicone-related compounds.

Compound	Formula	Mol. weight	Melting point (°C)	UV absorbance (nm)
Acetoxy-dihydrovermistatin	C_20_H_20_O_8_	388.1160	undetermined	210, 246, 305
Actofunicone	C_21_H_22_O_9_	418.1264	-	208, 246, 316
6-Demethylvermistatin	C_17_H_14_O_6_	314.0787	195–196	
Deoxyfunicone	C_19_H_18_O_7_	358.1053	127.5–128.5	220, 249, 314
14,15-Dihydrovermistatin	C_18_H_18_O_6_	330.1103	143–145	210, 245, 303
9,14-Epoxy-11-deoxyfunicone	C_19_H_18_O_8_	374.0975	166–167	203, 282
Funicone	C_19_H_18_O_8_	374.1004	176–178	245, 310, 368
Hydroxy-dihydrovermistatin	C_18_H_18_O_7_	346.1064	184–185	210, 248, 306
Hydroxyvermistatin	C_18_H_16_O_7_	344.0887	226–227	
Isofunicone	C_19_H_18_O_8_	374.1004	215–218	249, 285, 293
Methoxyvermistatin	C_19_H_18_O_7_	358.1053	198–199	
3-O-Methyl-5,6-epoxyfunicone	C_20_H_20_O_9_	404.1107	undetermined	210, 312
3-O-Methylfunicone	C_20_H_20_O_8_	388.1160	185–187	249, 293
Penicidone A	C_18_H_18_NO_5_	328.1179	undetermined	206, 237
Penicidone B	C_17_H_16_NO_5_	314.1023	undetermined	209, 239
Penicidone C	C_19_H_20_NO_6_	358.1285	undetermined	215, 253
Penisimplicissin	C_16_H_14_O_6_	302.0790	185–186	210, 247, 305
Rapicone	C_17_H_16_O_7_	332.0896	162–163	244, 314
Vermistatin	C_18_H_16_O_6_	328.0950	213–214	210, 262, 302

**Table 2:** Conditions for production, extraction and purification of funicone-related compounds.

Compound	Species	Reference	Substrate *	Conditions of growth	Extraction	Purification
Acetoxy-dihydrovermistatin	*P. simplicissimum*	6	Moist rice	21 d, 25°C	CH_2_Cl_2_-MeOH; EtOAc	CC; LPLC; HPLC
Actofunicone	*T. flavus*	10	Medium E	5 d, 27°C, AG	Me_2_CO; EtOAc	MPLC; HPLC
6-Demethylvermistatin	*Guignardia* sp.	22	Medium F1	25 d, 28°C	EtOAc	CC
Deoxyfunicone	*Penicillium* sp.	8	Medium C	6 d, 25°C, AG	EtOAc	CC
	*Penicillium* sp.	9	AD2	21 d, 22°C, AG	MeEtCO	CC; HPLC; TLC
	*T. flavus*	10	Medium E	5 d, 27°C, AG	Me_2_CO; EtOAc	MPLC; HPLC
14,15-Dihydroverm-istatin	*P. simplicissimum*	6	Moist rice	21 d, 25°C	CH_2_Cl_2_-MeOH; EtOAc	CC; LPLC; HPLC
	*E. euglaucum*	11	Wickerham	21 d, RT	EtOAc	CC
9,14-Epoxy-11-deoxy-funicone	*T. flavus*	5	Moist barley grains	21 d, 25°C	CH_2_Cl_2_-MeOH; EtOAc	CC; LPLC
Funicone	*P. funiculosum* (?)	3	Medium A	4 d	Et_2_O	TLC
	*T. flavus*	5	Moist barley grains	21 d, 25°C	CH_2_Cl_2_-MeOH; EtOAc	CC; LPLC
	*P. simplicissimum*	6	Moist rice	21 d, 25°C	CH_2_Cl_2_-MeOH; EtOAc	CC; LPLC; HPLC
Hydroxy-dihydrovermistatin	*P. simplicissimum*	6	Moist rice	21 d, 25°C	CH_2_Cl_2_-MeOH; EtOAc	CC; LPLC; HPLC
	*T. thailandiasis*	20	Moist rice	30 d, 28°C	EtOAc; CHCl_3_	CC
Hydroxyvermistatin	*Guignardia* sp.	21	Medium F	30 d, RT	EtOAc	CC; TLC
Isofunicone	*Penicillium* sp.	7	Medium D	21 d, 24°C, ST	EtOAc	CC
Methoxyvermistatin	*P. simplicissimum*	6	Moist rice	21 d, 25°C	CH_2_Cl_2_-MeOH; EtOAc	CC; LPLC; HPLC
	*Guignardia* sp.	21	Medium F	30 d, RT	EtOAc	CC
3-O-Methyl-5,6-epoxyfunicone	*P. pinophilum*	14	PDB	21 d, 25°C, ST	CH_3_Cl; Me_2_CO-MeOH	TLC
3-O-Methylfunicone	*P. pinophilum*	12	PDB	21 d, 25°C, ST	CH_3_Cl; Me_2_CO-MeOH	TLC
Penicidones	*Penicillium* sp.	28	Medium G	20 d, 28°C	MeOH; EtOAc	CC
Penisimplicissin	*P. simplicissimum*	6	Moist rice	21 d, 25°C	CH_2_Cl_2_-MeOH; EtOAc	CC; LPLC; HPLC
	*T. thailandiasis*	20	Moist rice	30 d, 28°C	EtOAc; CHCl_3_	CC
Rapicone	*R. apiculatum*	13	PDB	21 d, 28°C, ST	CH_2_Cl_2_	CC; LPLC
Vermistatin	*T. flavus*	15	Medium B	5–6 d, 28°C, AG	CH_2_Cl_2_	TLC
		17	Czapek-Dox broth	30 d, 27°C, AG	EtOAc	CC; TLC
		10	Medium E	5 d, 27°C, AG	Me_2_CO; EtOAc	MPLC; HPLC
		5	Moist barley grains	21 d, 25°C	CH_2_Cl_2_-MeOH; EtOAc	CC; LPLC
	*M. fijiensis*	18	M-1-D/coconut	28 d, 26°C, AG, 12h	MeOH; EtOAc	TLC; HPLC
	*P. verruculosum*	19	Medium H	17 d, 24°C	EtOAc	CC
	*P. simplicissimum*	6	Moist rice	21 d, 25°C	CH_2_Cl_2_-MeOH; EtOAc	CC; LPLC; HPLC
	*E. euglaucum*	11	Wickerham	21 d, RT	EtOAc	CC
	*T. thailandiasis*	20	Moist rice	30 d, 28°C	EtOAc; CHCl_3_	CC
	*Guignardia* sp.	21	Medium F	30 d, RT	EtOAc	CC; TLC

* also see Appendix. Abbreviations: PDB, potato-dextrose broth; RT, room temperature; AG, cultures maintained in agitation; ST, cultures on stationary phase; 12h, 12-hour photoperiod; MeOH, methanol; EtOAc, ethyl acetate; Me_2_CO, acetone; MeEtCO, methyl ethyl ketone; CH_2_Cl_2_, dichloromethane; Et_2_O, diethyl ether; CHCl_3_, chloroform; CC, column chromatography; LPLC, low performance liquid chromatography; HPLC, high performance liquid chromatography; MPLC, medium performance liquid chromatography; TLC, thin-layer chromatography.

## Appendix: Synthetic Media Used for Production of Funicone-Related Compounds (Composition Per Litre, Except for Semisolid Medium G)

**Table:**

Medium A [3]	
Glucose	40 g
Ammonium tartrate	4.7 g
KH_2_PO_4_	1.02 g
MgSO_4_	0.51 g
KCl	0.51 g
FeSO_4_	0.01 g
Agar	
Medium B [15]	
Glucose	9%
NaNO_3_	0.2%
KH_2_PO_4_	0.1%
MgSO_4_·7H_2_O	0.05%
KCl	0.05%
FeSO_4_·7H_2_O	0.001%
pH = 6.3	
Medium C [8]	
Commercial sugar (saccharose ?)	4%
Corn steep liquor	2%
Medium D [7]	
Malt extract	20 g
Glucose	50 g
Peptone	3 g
Medium E [10]	
PDB	2.4%
Malt extract	0.5%
Mg_3_(PO_4_)_2_·8H_2_O	0.5%
Agar	0.1%
pH = 6.0	
Medium F [21]	
Glucose	10 g
Peptone	2 g
Yeast extract	1 g
NaCl	30 g
Medium F1 [22]	
Glucose	10 g
Peptone	2 g
Yeast extract	1 g
NaCl	2 g
pH = 7.0	
Medium G [28]	
Grain	7.5 g
Bran	7.5 g
Yeast extract	0.5 g
Sodium tartrate	0.1 g
FeSO_4_·7H_2_O	0.01 g
Sodium glutamate	0.1 g
Pure corn oil	0.1 mL
Water	30 mL
Medium H [19]	
Carrot extract	?
Glucose	50 g
NaNO_3_	3 g
KH_2_PO_4_	1 g
MgSO_4_·7H_2_O	0.5 g
KCl	0.5 g
FeSO_4_·7H_2_O	0.01 g
pH = 4.5	
AD-2 [9]	
Glucose	150 g
Glycerol	20 g
Yeast extract	4 g
NaNO_3_	1 g
Sodium glutamate	3 g
Na_2_HPO_4_	0.5 g
MgSO_4_·7H_2_O	1 g
Trace element solution	1 mL
CaCO_3_	8 g
pH = 7.0	
M-1-D/coconut [18]	
Inositol	5 g
Thiamine	0.5 g
Biotin	0.5 g
Coconut water	12 mL
Wickerham [11]	
Glucose	10 g
Bacto-peptone	5 g
Yeast extract	3 g
Malt extract	3 g
pH = 7.3	
